# Design of a Dual-Drug Delivery System for Local Release of Chlorhexidine and Dexketoprofen

**DOI:** 10.3390/polym17131771

**Published:** 2025-06-26

**Authors:** Vicente Esparza-Villalpando, Amaury Pozos-Guillén, Ángel Antonio Vértiz-Hernández, Jose Vega-Baudrit, Daniel Chavarría-Bolaños

**Affiliations:** 1 Stomatology Department, Universidad Autónoma de Aguascalientes, Aguascalientes 20100, Mexico; vicente.esparza@edu.uaa.mx; 2 Basic Science Laboratory, Facultad de Estomatología, Universidad Autónoma de San Luis Potosí, San Luis Potosi 78290, Mexico; apozos@uaslp.mx; 3 Unidad Académica Multidisciplinaria Región Altiplano, Universidad Autónoma de San Luis Potosí, Matehuala 78700, Mexico; antonio.vertiz@uaslp.mx; 4 Laboratorio Nacional de Nanotecnología, Centro Nacional de Alta Tecnología, San Jose 10109, Costa Rica; jvegab@gmail.com; 5 Dentistry Graduate Program, Universidad de Costa Rica, San Jose 11501, Costa Rica

**Keywords:** drug delivery system, chlorhexidine, dexketoprofen, microspheres, hydrogels

## Abstract

Background: This study developed and characterized a novel drug delivery system (DDS) for potential use in oral surgery, combining poly(lactic-co-glycolic acid) (PLGA) microspheres loaded with chlorhexidine (MS-CHX) and a polyethylene glycol (PEG)-based hydrogel containing dexketoprofen (HG-DXT). Methods: MS-CHX was synthesized using a double emulsion evaporation method, while HG-DXT was formulated from a PEG blend. The components were combined in a 2:1 ratio to create the MS-CHX/HG-DXT DDS. Characterization techniques included differential scanning calorimetry (DSC), thermogravimetric analysis (TGA), scanning electron microscopy (SEM), Fourier transform infrared spectroscopy (FTIR), and energy-dispersive X-ray spectroscopy (EDS). Antibacterial activity was evaluated using disk diffusion assays against *E. faecalis*, *E. coli*, *S. aureus*, and *C. albicans*. Biocompatibility was assessed with MTS, and drug release was measured via high-performance liquid chromatography (HPLC) in vitro. Results: CHX-loaded microspheres showed spherical morphology, stability above 37 °C, and antimicrobial efficacy. HG-DXT demonstrated good biocompatibility (80% of cell viability) and stable physicochemical properties (stability at 50-day storage). The DDS exhibited a biphasic release: an initial burst of dexketoprofen for analgesia, followed by sustained release of chlorhexidine for antimicrobial protection. Conclusions: This novel dual-action DDS showed promising characteristics and a favorable release profile, supporting its potential as a therapeutic alternative for post-operative pain and infection control in oral surgical procedures.

## 1. Introduction

Certain surgical approaches aim to eliminate infectious foci that cannot be controlled by routine treatments, such as periodontal surgery or endodontic microsurgery. The latter is a promising alternative with high success rates [1] and provides an opportunity to preserve teeth with persistent periapical lesions when conventional endodontic retreatment has failed due to persistent infections [2]. These procedures differ from other surgical interventions, such as third molar extractions or implant placements, where preoperative infection is typically not a primary concern. In such cases, clinicians face a dual microbial challenge: managing the infectious focus locally while also preventing postoperative wound infections. Furthermore, the surgical procedure itself and the resulting wound inherently induce local inflammation and pain [3], complicating postoperative management.

A wound is defined as a disruption of normal anatomical structure and function [4]. Surgical wounds resulting from surgical interventions trigger an inflammatory response, leading to the release of various chemical mediators, including prostaglandins [5]. Non-steroidal anti-inflammatory drugs (NSAIDs) inhibit the active sites of cyclooxygenases (COXs), thus reducing the release of prostaglandins. At the local level, NSAIDs act by activating peripheral mechanisms, allowing for the effective control of pain originating from the surgical wound [6]. Local NSAIDs primarily target the application sites [7], achieving clinical effectiveness with minimal dosage (less than 5%) compared to oral administration [8]. Among topical formulations, the best results have been observed with ibuprofen, ketoprofen, and diclofenac [9]. Dexketoprofen trometamol (DXT) is an NSAID belonging to the aryl propionic acid derivatives family. The S (+) enantiomer of the compound ketoprofen is well-known for its analgesic and anti-inflammatory effects [10,11].

The healing of surgical wounds can be impeded by various factors, and surgical site infections (SSIs) always pose a potential risk, as all wounds are inherently contaminated regardless of their size or location. The manifestation of SSIs depends on factors such as the virulence, quantity, and type of microorganisms involved, as well as the blood supply to the site and the patient’s inherent resistance [12]. To mitigate the risk of infection, adjunctive measures such as pre-surgical antiseptic showers and site cleaning are strongly recommended and supported by robust evidence [13].

Topical biocides find extensive use as disinfectants and antiseptics, playing a crucial role in surgical cleaning protocols aimed at preventing infections. Unlike antibiotics, biocides have a broader spectrum of action and employ multiple mechanisms [14]. Among the most employed biocides for preventing SSIs are chlorhexidine, triclosan, povidone-iodine, and alcohol, among others [15]. Chlorhexidine digluconate (CHX), derived from chlorobenzenes, is a frequently used antiseptic in various surgical protocols. Its mechanism of action involves the ionic binding between the cationic CHX and the anionic cell wall of microorganisms, forming extra-microbial complexes [16]. This mechanism imparts antibacterial, bacteriostatic, and antifungal properties against a wide range of Gram-positive and Gram-negative strains [17].

In endodontic surgery, the physiological response typically involves an inflammatory phase accompanied by acute pain and the potential risk of SSI. As mentioned earlier, these clinical challenges are often addressed using strategies such as systemic analgesic prescriptions and the prophylactic administration of oral antibiotics [13]. However, systemic administration of these drugs can lead to certain disadvantages, including the need for high doses, multiple administrations, uncertain bioavailability, exposure of the drugs to distant body compartments, and potential side effects [18]. To mitigate these issues, drug delivery systems (DDS) can be employed to ensure controlled release of therapeutic agents directly at the site of SSI. Multiple promising solutions have been developed, such as hydrogels with novel applications [19], devices for periodontal treatment [20], nanoparticles, nanolayers, films, and scaffolds [21] for oral diseases. However, the clinical use of these solutions requires multiple research stages; therefore, it is important to develop new systems to increase the likelihood of reaching the final stage for DDS to address dental problems. The objective of this study was to synthesize and characterize a DDS consisting of poly(lactic-co-glycolic acid) (PLGA) microspheres (MS) loaded with CHX (MS-CHX), incorporated into a polyethylene glycol (PEG) hydrogel loaded with DXT (HG-DXT).

## 2. Materials and Methods

This project was authorized by the Institutional Ethics Committee, under the code CEI-FE-003-19.

### 2.1. MS-CHX/HG-DXT DDS Preparation

#### 2.1.1. PLGA MS-CHX-Loaded Fabrication

The MS were fabricated using the double emulsion evaporation method. CHX at a concentration of 20%, PLGA (50:50; molecular weight range ∼66,000–110,000), dichloromethane (DCM), and polyvinyl alcohol (PVA, 87–90%; molecular weight range ∼30,000–70,000) were obtained from Sigma–Aldrich^®^ (St. Louis, MO, USA). Briefly, 200 µg of PLGA was dissolved in 4 mL of DCM, which was mixed for 2 min. Subsequently, 500 µL of a 2% CHX solution (equivalent to 10 mg of CHX) in water (MS-CHX) or 500 µL of water (MS-Blank) was added. This emulsion was vortexed for 3 min and ultrasonicated at a potency level of 3 (Microson^®^ XL, Qsonica, LQsonica, LLC., Newtown, CT, USA for 5 min. Then, the emulsion was added to a 2% PVA water solution (100 mL) and mixed for 2 h using an IKA^®^ RW 20 digital mixer (IKA^®^ Works, Inc., Staufen, Germany). Finally, the MS were obtained by filtration using filter paper no. 1 (Whatman^®^ Filter Papers, GE Healthcare, Little Chalfont, UK) and subsequently dried for 24 h at room temperature.

#### 2.1.2. PEG HG-DXT-Loaded Preparation

**Figure 1:**
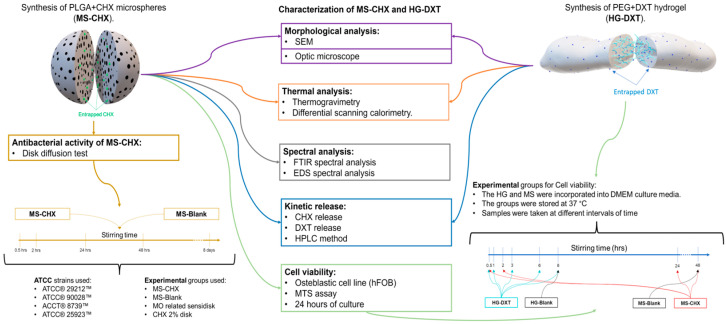
Graphical description of the experimental methods.

The HG was created by physically combining two types of PEG with different molecular weights (Mw). DXT (STEIN labs^®^, San Jose, Costa Rica) and PEG (MW: 400 and 4000) were obtained from Sigma–Aldrich^®^ (St. Louis, MO, USA). To create the HG, 25 mg of DXT was dissolved in 812.5 mg of PEG 400 and stirred the mixture on a magnetic hot plate (C-MAG HS7, IKA®, Staufen, Germany) for 6 min at a temperature ranging between 100 and 103 °C. Afterward, 162.5 mg of PEG with a molecular weight of 4000 was added and stirred until the polymers dissolved completely, resulting in a crystalline appearance. Finally, the mixture was allowed to cool at room temperature. The blank hydrogel (HG-Blank) contains only a mixture of PEG polymers and was prepared identically, without the addition of DXT. The description of experimental groups is shown in Table 1 and Figure 1.

### 2.2. Morphological Characterization of MS-CHX, HG-DXT, and MS-CHX/HG-DXT DDS

A morphological characterization to assess the shape and size of MS-CHX, HG-DXT, and the MS-CHX/HG-DXT DDS was conducted by scanning electron microscopy (SEM) (JEOL Ltd., Tokyo, Japan) and optical microscopy. In the SEM imaging process, the MS were first dried, fixed to adhesive carbon tape, and then coated with a layer of gold via sputtering (BAL-TEC, SCD 005 Sputter Coater, Scotia, NY, USA). The accelerating voltage for SEM was set at 10 kV. Subsequently, combinations of MS-CHX and HG-DXT were prepared to create the MS-CHX/HG-DXT DDS in two distinct mass-to-mass proportions, namely 1:1 and 1:2. The morphological characteristics of both the HG and the combination of MS and HG (DDS) were assessed using optical microscopy (Leica, BME, Leica Microsystems, Wetzlar, Germany) (Figure 1).

### 2.3. Thermal Characterization

Thermogravimetric analysis (TGA) was conducted using the TGA 550 system (TA Instruments, New Castle, DE, USA). The operational parameters included a starting temperature of 25 °C and a final temperature of 1000 °C, with a heating ramp of 20 °C per minute, all conducted under a flow rate of 90 mL/min in a nitrogen atmosphere. Differential scanning calorimetry (DSC) was performed using the DSC 250 system (TA Instruments, New Castle, DE, USA). The parameters consisted of a starting temperature of 20 °C and a final temperature of 120 °C, with a heating ramp of 10 °C per minute, and a flow rate of 50 mL/min in a nitrogen atmosphere. Each sample used for TGA and DSC analysis weighed approximately 2–4 mg. For the MS, the analyzed samples included MS-Blank, Pure PLGA, CHX 20% lyophilized, and MS-CHX. In addition, HG-Blank, Pure PEG, pure DXT, and HG-DXT were analyzed. Furthermore, the combinations of MS-CHX/HG-DXT DDS in proportions of 1:1 and 1:2 were examined (Figure 1).

### 2.4. Spectral Analysis of MS

Fourier transform infrared spectroscopy (FTIR) spectra for MS-Blank and MS-CHX were obtained using an FTIR device (Nicolet™ iS™ 50 spectrometer Thermo Fisher Scientific, Waltham, MA, USA). This analysis aimed to identify any variations in spectral peaks within the MS, and to confirm the presence of CHX in the MS. The MS (in powdered form) were placed in the FTIR detector and compared with the spectra of pure PLGA and lyophilized CHX. Additionally, surface elemental analysis was conducted using Energy-dispersive X-ray spectroscopy (EDS). Both loaded and unloaded MS were affixed to adhesive carbon tapes and analyzed using the EDS System with a 30 eV energy pass (JEOL Ltd., Tokyo, Japan).

### 2.5. Antibacterial Evaluation of MS

To assess the antibacterial activity of the MS, the disk diffusion test was employed. For this test, microorganisms including *Enterococcus faecalis* (*E. faecalis*) ATCC^®^ 29212™, *Candida albicans* (*C. albicans*) ATCC^®^ 90028™, *Escherichia coli* (*E. coli*) ACCT^®^ 8739™, and *Staphylococcus aureus* (*S. aureus*) ATCC^®^ 25923™ were selected. These microorganisms were tested with the following experimental groups: MS-CHX, MS-Blank, CHX 2%, and strain-specific sensi-disks (containing amoxicillin + clavulanic acid, ceftriaxone, and voriconazole). All microorganisms were cultured following the ATCC culture guidelines *provided by the manufacturer. To prepare the MS-CHX solution, 34.2 mg of MS were* mixed with 0.5 mL of pure water (this concentration was selected solely for assessing the antimicrobial activity of CHX loaded in the MS) in 1.5 mL tubes. The mixture was stirred at various intervals (0.5, 2, 24, 48 h, and 8 days). On the day of the experiment, the tubes were centrifuged (for 5 min at 2000 rpm), and the supernatant was collected. After seeding the microorganisms in agar Petri dishes, the experimental groups were applied to 6 mm paper disks and incubated for 48 h at 37 °C. Subsequently, the inhibition halo was measured (Figure 1).

### 2.6. Cytotoxicity Evaluation of the MS-CHX and HG-DXT

To evaluate the cytotoxic effect of the MS-CHX and HG-DXT individually, an MTS was used (CellTiter 96® AQueous Non-Radioactive Cell Proliferation Assay (MTS) (Promega Corporation, Madison, WI, USA). Briefly, osteoblastic cell lines (hFOB 1.19, ATCC^®^) were cultivated using culture medium low-glucose DMEM (BioWest, Nuaillé, France) at 5% of BFS (bovine fetal serum) and 1% antibiotic; the cells were cultured in Petri boxes for 24 h, and after that, the adhered cell was detached using an enzymatic process with trypsin (TrypLE™ Express (Thermo Fisher Scientific, Waltham, MA, USA). In a 96-well cell culture plate, 20,000 cells were added to each well. For the MS cytotoxic effect, 410 mg of MS (MS-CHX and MS-Blank) was incorporated in 6 mL of culture media. This solution was stirred at 37 °C for 0.5, 2, 24, and 48 h, as well as at 8 days; at each interval, 600 µL of the supernatant was taken. For the HG cytotoxic effect, 500 mg was incorporated in 5 mL of culture media; this solution was stirred at 37 °C for 0.5, 1, 3, 6, and 8 h. The experimental groups (*n =* 5 for each group) used were as follows: for the MS-CHX, samples were taken at 0.5, 2, 24, 48 h, and 8 days of stirring, for MS-Blank, samples were taken only at 48 h of stirring; for HG-DXT, samples were taken at 0.5, 1, 3, 6, and 8 h of stirring; for HG-Blank, samples were taken only at 8 h; for the dead control H_2_O_2_ was used; for the live control, only culture media was used; and for the drug control, 0.2% CHX and 2.5% DXT were used (Figure 1). An additional direct-contact evaluation was performed for all groups including MS-CHX/HG-DXT and MS-Blank/HG-Blank in 1:2 proportion (Figure 1); 6 mg of each compound were added in each well with 20,000 cells (*n =* 5). The cultured conditions for all experiments and groups were 24 h at 37 °C, with 100% humidity and 5% CO_2_. For the MTS, the protocol was based on the manufacturer’s suggested reference [17]. The cell viability of the experimental groups was calculated by the following formula:

$$Cell\;Viabilidy(\%)={{(Group}_{i}\times 100)/\bar{x}}_{Live\;control}$$

### 2.7. Kinetics of CHX and DXT Release and Quantification from MS-CHX/HG-DXT DDS

For the quantification of CHX and DXT released from the MS-CHX/HG-DXT DDS, high-performance liquid chromatography (HPLC) using an Altus A-10 system (PerkinElmer, Waltham, MA, USA) equipped with a UV-VIS detector was employed. This system included an autosampler and column thermostat. To establish the chromatographic conditions for each drug and their combination, CHX solution (H_2_O 20%, Sigma–Aldrich^®^, St. Louis, MO, USA) and pure DXT (STEIN labs^®^, San Jose, Costa Rica) were used as standard samples. The stability of the samples after a 50-day storage period during which they were frozen at −4 °C was assessed. The variance coefficient ranged from 0.99% to 4.19% for CHX and from 1.1% to 10.9% for DXT. The following chromatographic conditions were set: Column: Thermocienfic Acclaim 300 (3 µm, 4.6 × 50 mm) C18, mobile phase: A: Acetonitrile, B: H_2_O + H_3_PO_4_, A + B (77:23), flux: 1.8 mL/min, injection volume: 10 µL, column temperature: 20 °C, wavelength: 239 nm (Absorbance), running time: 5.5 min, retention time: 1.0 min for CHX, 3.2 min for DXT, detection range: 9.1 µg/mL to 364 µg/mL. The kinetics release of CHX and DXT was performed for 35 days, using a 12 mm Transwell^®^ inserts with polytetrafluoroethylene (PTFE) membrane insert (Corning® #3495, Corning Inc., Corning, NY, USA). The MS-CHX and HG-DXT, and their combination in proportions 1:1 and 1:2 were placed inside the inserts (10 mg, with 1 mL of pure water (HPLC suitable water (WX0004, Supelco®, Bellefonte, PA, USA) for triplicate (Figure 1) and agitated using an orbital shaker at 37 °C; at each time point (0.5, 1, 4, 8 h, 1, 3, 5, 7, 10, 14, 18, 22, 26, 29, 32, and 35 days) 900 µL was taken from the inserts, and new pure water was added until the next sampling, maintaining a pH of 3.

### 2.8. Statistical Analysis

The quantitative data are shown as mean and standard deviation (sd), the normality of the data was assessed using the Shapiro–Wilk test, and the homogeneity of variances was evaluated with Levene’s test. The comparison between groups was performed with ANOVA, Kruskal–Wallis, *t*-test, or Wilcoxon sum-rank, depending on the data distribution. The post hoc analysis was made with planned contrast ANOVA, Tukey multiple comparisons test, and Siegel & Castellan method for non-parametric multiple comparisons. The confidence of the statistical test was set at 95% with 80% of statistical power. All analyses were performed in R version 3.5.2.

## 3. Results

### 3.1. Characterization of HG and MS

**Figure 2:**
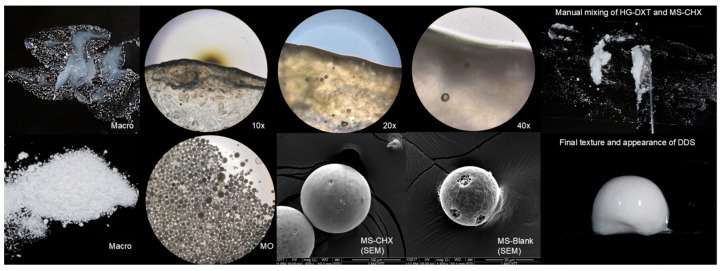
Physical characterization of the MS-CHX, HG-DXT and MS-CHX/HG-DXT DDS.

The MS exhibited a macroscopic appearance of white, dry powder (Figure 2). Under microscopic evaluation (10×), the MS displayed varying sizes and spherical shapes. The loaded MS (MS-CHX) had an average size ranging from ~100 µm to 250 µm, while the MS-Blank ranged from ~50 µm to 150 µm. SEM evaluation revealed that MS-CHX had a regular spherical shape with a uniform surface and the presence of pores. MS-Blank, on the other hand, displayed an irregular surface with larger pores compared to MS-CHX. Additionally, the surface of MS-Blank exhibited depressions and a “raisin-like” appearance (Figure 2). The macroscopic appearance of HG was a semi-fluid, petroleum jelly-like gray material with an aqueous appearance and bubbles present within the material. Crystalline formations were not observed in the final mixture. HG-Blank and HG-DXT did not exhibit morphological differences. The MS-CHX/HG-DXT DDS appeared as a white semi-fluid gel with a physical texture that was easy to handle and manipulate (Figure 2).

### 3.2. Thermal Characterization of MS-CHX, HG-DXT, and DDS

**Figure 3:**
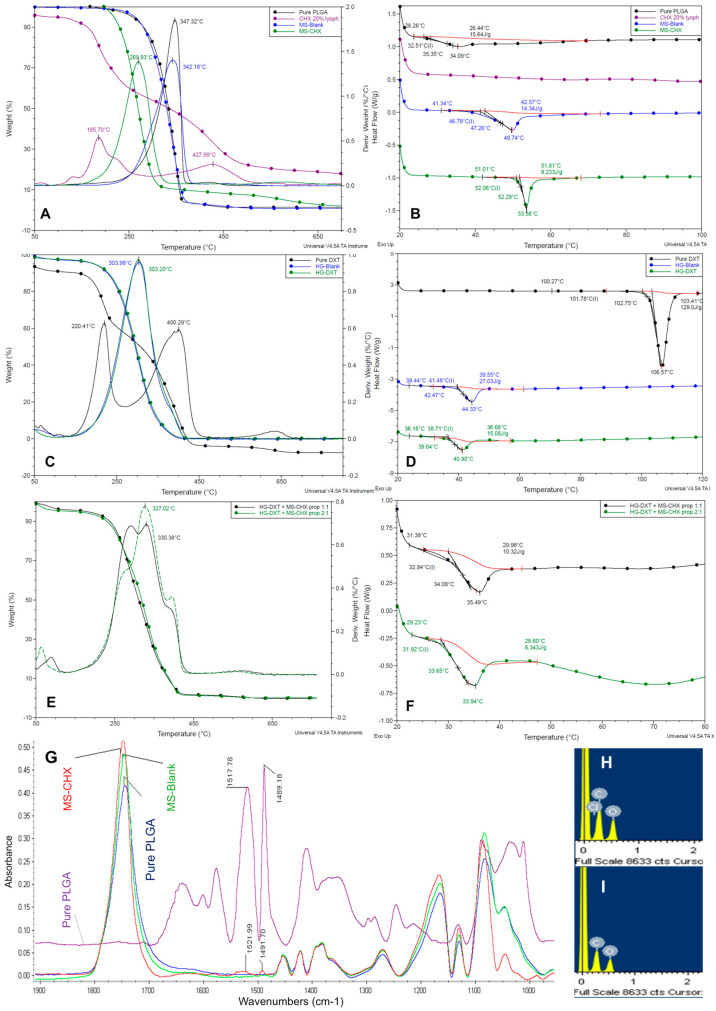
Thermal and spectral characterization of MS-CHX, HG-DXT, and MS-CHX/HG-DXT DDS. (**A**,**B**) Thermal behavior of the MS-CHX, TGA, and DSC, respectively. (**C**,**D**) Thermal behavior of the HG-DXT, TGA, and DSC, respectively. (**E**,**F**) Thermal behavior of the MS-CHX/HG-DXT DDS, TGA, and DSC, respectively. (**G**) FTIR spectra of the MS-CHX. (**H**) EDS spectra of the MS-CHX. (**I**) EDS spectra of the MS-Blank.

The thermal characterization is shown in Figure 3A–F. TGA results are presented as follows: MS-CHX (Figure 3A), HG-DXT (Figure 3C), and MS-CHX/HG-DXT DDS (Figure 3E). The inflection point (Tp) calculated by the first derivative of mass loss curve (%/°C) of MS-Blank (blue line) and pure polymer PLGA (black line) exhibit the same shape and practically the same temperature peak at 342.18 °C and 347.32 °C, respectively, while pure CHX (purple line) showed the first signal at 185.07 °C. MS-CHX (green line) shifted to a lower temperature at 269.93 °C, showing a thermal profile between the two main components. The Tp of HG-Blank (blue line) and pure HG-DXT (green line) exhibit the same shape and practically the same IP, 303.98 °C and 303.2 °C, respectively. The pure DXT (black line) shows two peaks at 220.41 °C and 400.29 °C (Figure 3C). Finally, the Tp of the DDS at a 1:1 ratio (black line) shows one defined peak at 330.38 °C, while the 2:1 ratio (green line) shows one peak at 327.02 °C (Figure 3E).

DSC results are presented for MS-CHX (Figure 3B), HG-DXT (Figure 3D), and MS-CHX/HG-DXT DDS (Figure 3F). The endothermal change in the pure PLGA (black line) showed a change in flow heat that started at 28.26 °C to 35.35 °C (Tg = 32.51 °C). For the CHX (purple line), it was not possible to identify any change in the flow heat from 20 to 120 °C. For the MS-Blank (blue line), an endothermal change was observed at 49.74 °C, starting at 41.34 °C (Tg = 49.74 °C). For MS-CHX (green line), the endothermal change is shifted to the right, at 53.58 °C, starting at 51.01 °C (Tg = 52.06 °C). The endothermal peak shapes of MS-CHX and MS-Blank are different, showing a more symmetrical shape for MS-CHX than the MS-Blank signal. This suggests a better molecular organization of the loaded MS. All the MS showed thermodynamical stability at body temperature (Figure 3B).

For the HG samples, the pure DXT (black line) showed a melting point at 106.57 °C, the HG-Blank (blue line) showed an endothermal signal that starts at 39.44 to 42.47 °C (Tg = 41.48 °C), while the HG-DXT (green line) showed that the endothermal change starts between 36.18 and 39.64 °C (Tg = 38.71 °C) (Figure 3D). This behavior indicates thermodynamic instability at body temperature. The same behavior is observed for the DDS at the 1:1 ratio (black line) with a change in flow heat starting at 31.38 to 34.08 °C (Tg = 32.84 °C), while att the 2:1 ratio (green line) the change in flow heat starts at 29.23 to 33.65 °C (Tg = 31.92 °C) (Figure 3F).

### 3.3. Spectral Analysis of MS-CHX

The FTIR analysis indicated that MS-CHX, MS-Blank, and pure PLGA exhibited similar spectra, except for two signal peaks exclusive to MS-CHX. These two signals corresponded to bands at 1521 cm^−1^ and 1491 cm^−1^, signifying the presence of C=N (amine groups) characteristic of CHX, in addition to the 1668 cm^−1^ and 1598 cm^−1^ bands, which corresponded to the amine groups found in CHX (bands 1517 and 1489). This is visually represented in the purple and red spectra (Figure 3G). These results provide strong evidence of the presence of CHX in MS-CHX. In the EDS spectra, both MS-CHX (Figure 3H) and MS-Blank (Figure 3I) exhibited the presence of carbon (C) and oxygen (O), which are constituents of the PLGA polymer. However, only MS-CHX showed the presence of chlorine (Cl), suggesting the presence of CHX.

### 3.4. Antibacterial Effect of MS

The inhibition areas were observed in the MS-CHX groups across all bacterial strains. Notably, this zone increased with prolonged stirring time. Conversely, the MS-Blank group did not exhibit any inhibition zones for any of the bacterial strains. Comparatively, CHX 2% and sensi-disk groups displayed larger inhibition zones in comparison to MS-CHX. Specifically, for the *E. faecalis* group, MS-CHX exhibited an effect comparable to CHX 2% and ceftriaxone sensi-disk by day 8 (*p* > 0.05). In the case of the *C. albicans* group, MS-CHX demonstrated a smaller antimicrobial effect (*p* < 0.05) when compared with Voriconazole and CHX 2%. However, the antimicrobial effect was still evident and categorized as susceptible, with an inhibition zone diameter exceeding 17 mm. For *E. coli*, after 8 days of stirring, MS-CHX exhibited a slightly smaller antimicrobial effect (*p* < 0.05) compared to Ceftriaxone and CHX 2%. However, the antimicrobial effect remained present and fell into the intermediate category, with an inhibition zone diameter ranging between 13 and 15 mm. Similarly, in the case of the *S. aureus* group, after 8 days of stirring, MS-CHX displayed a slightly smaller antimicrobial effect (*p* < 0.05) than CHX 2%. Nevertheless, it was slightly larger than the effect observed with Amoxi/Clav. These results are summarized in Table 2.

### 3.5. Cytotoxic Effect of the MS-CHX and HG-DXT

**Figure 4:**
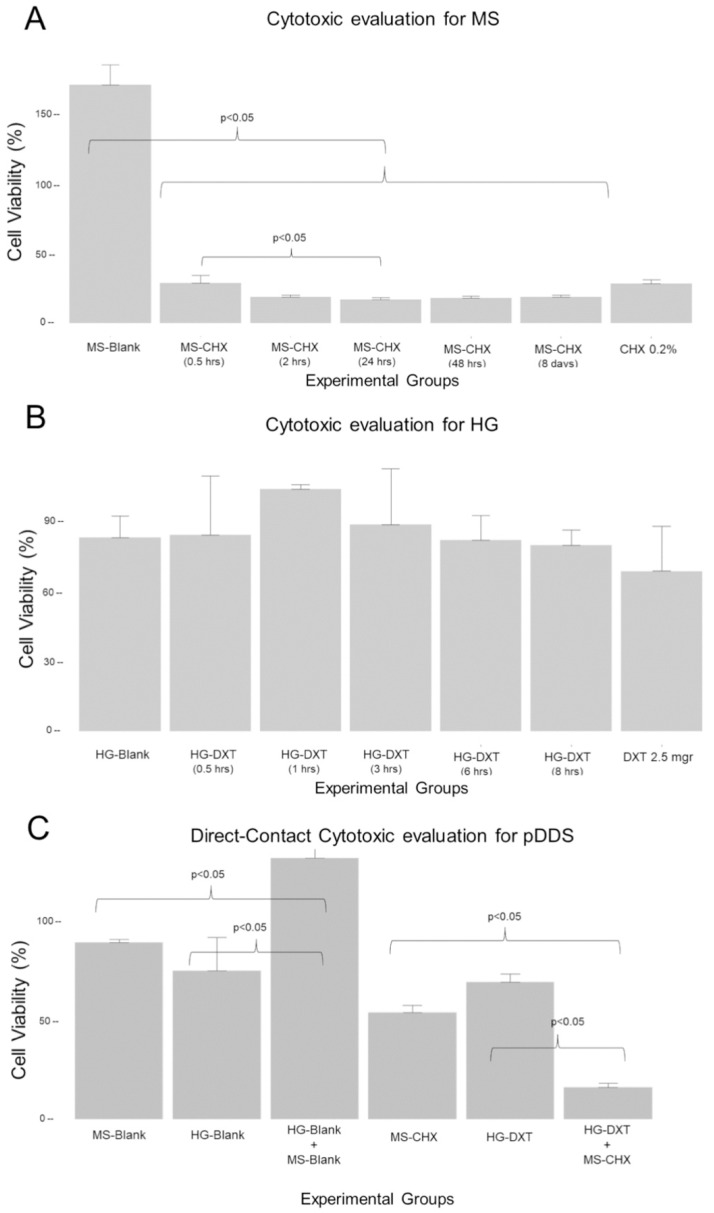
Data for the cytotoxic evaluation of the MS and HG, and DDS direct contact. (**A**) Shows the cell viability evaluation for MS-CHX; there are significant differences (*p* < 0.05) between the MS-Blank and all MS-CHX groups, as well as between the MS-CHX at 30 min of stirring and the MS-CHX group at 24 h of stirring. (**B**) Shows the cell viability evaluation for the HG-DXT, revealing no evidence of the cytotoxic effect of the hydrogel groups. (**C**) Shows the cell viability evaluation for the direct-contact assay; the data show evidence of the cytotoxic effect of the MS-CHX and DDS.

To evaluate the difference in cell viability between MS-CHX groups, the Kruskal–Wallis test was used, with the Shapiro–Wilk test W = 0.52 and *p*-value < 0.001, and the Levene test with F = 3.52 and *p* = 0.01. The viability difference between groups was significant with chi-squared test result = 27.9, df = 6, and *p*-value < 0.0001. Multiple comparisons were made with the Siegel & Castellan method, with a significant difference (*p* < 0.05) between the MS-Blank and MS-CHX groups and MS-CHX (0.5 h) and MS-CHX (24 h). These findings are graphically presented in Figure 4A. A one-way ANOVA test was used to evaluate the difference in cell viability between HG-DXT groups, with Shapiro–Wilk test W = 0.98 and *p*-value = 0.697, and Levene test with F = 1.18 and *p* = 0.34. The test results indicated no significant variation among the groups (F = 2.09, df = 6.28, *p*-value = 0.08). These results are represented in Figure 4B. A one-way ANOVA test was used to evaluate the difference in cell viability between groups when the materials were in the direct-contact assay. The Shapiro–Wilk test was W = 0.94 and *p*-value = 0.11, and the Levene test was F = 1.08 with *p* = 0.39. The test results showed significant variation among the groups (F = 119.7, df = 5.24, *p*-value < 0.001), with Tukey’s direct comparisons revealing significance between groups, except for HG-Blank vs. MS-Blank and HG-Blank vs. HG-DXT. These results are represented in Figure 4C. Notably, direct contact with the MS-CHX and the MS-CHX/HG-DXT system exhibited a potential cytotoxic effect, underscoring the importance of avoiding such contact. In contrast, HG-DXT and MS-Blank/HG-Blank demonstrated no cytotoxic effect on the tested cells, signifying their safety for cell viability. The results are summarized in Table 3.

### 3.6. Kinetics Release and Quantification of CHX and DXT from the DDS

**Figure 5:**
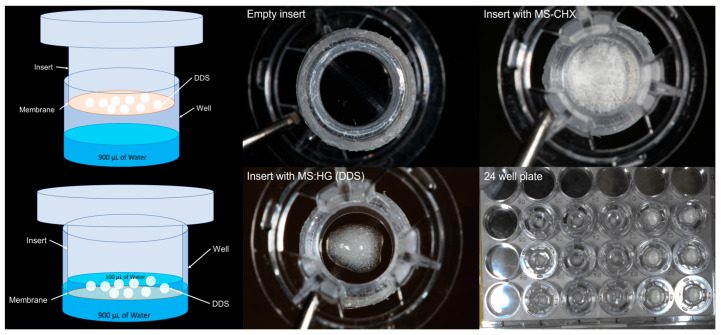
Protocol for the kinetic release experiment.

The encapsulation efficiency and drug loading of MS-CHX were determined by measuring the amount of CHX based on its density (1.06 g/mL). During the MS elaboration process, 10.6 mg of CHX was combined with 200 mg of PLGA. This process exhibited approximately 60.8% efficiency, resulting in an average MS yield of approximately 110 mg. As a result, the theoretical content of CHX in 10 mg of MS is approximately 6.444 mg, which translates to a theoretical concentration of CHX at around 5.8% (~585.89 µg). For HG-DXT, the theoretical concentration of DXT was set at 2.5%, meaning that in 10 mg of HG-DXT, the amount of DXT would be approximately 250 µg. In Figure 5, the experimental conditions for the release assay are presented. The linearity for CHX and DXT drugs was found to be R^2^ = 0.9955 and 0.9975, respectively. The mass for MS-CHX, HG-DXT, and their combinations in proportions of 1:1 and 2:1 used for the experiment was 10 mg, with *n* = 3 (Table 4). The initial signal in the HPLC detector (FS) for CHX in MS-CHX appeared around 72 h, while for DDS-1:1 and DDS-2:1, it occurred approximately at 120 h. In the case of DXT, the FS in HG-DXT was detected at about 0.5 h, whereas for DDS-1:1 and DDS-2:1, it was observed at 24 and 0.5 h, respectively (Table 4). The kinetic characterization was made using the Korsmeyer–Peppas equation $M_{t}/M_{\infty }={Kt}^{n}$, and the table reported by Dash et al., 2010 [22].

**Figure 6:**
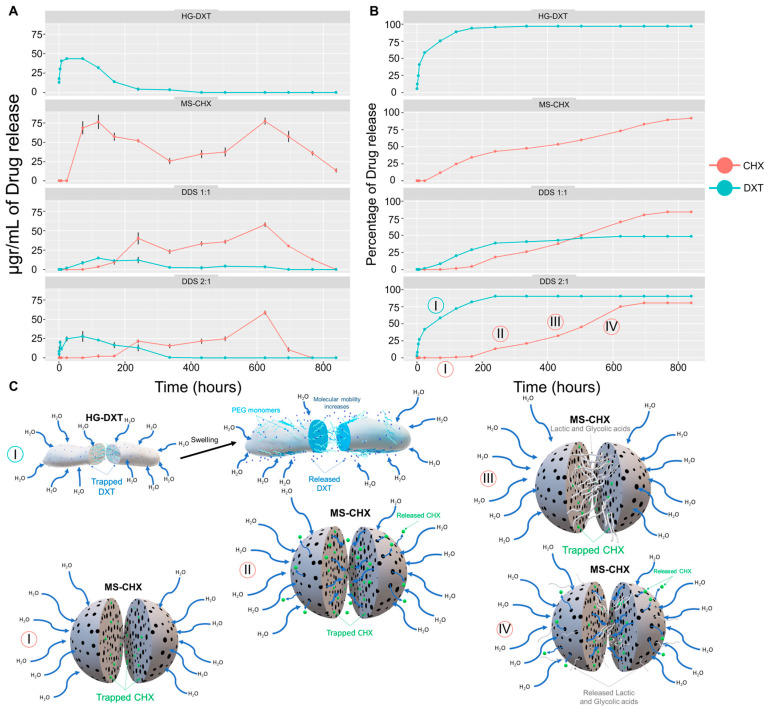
DXT and CHX kinetic release from DDS. (**A**) Shows the concentration of drug release at each interval time of the CHX and DXT released from the HG-DXT, MS-CHX, and DDS. (**B**) Shows the percentage of drug release from the same groups. (**C**) Shows the four stages explaining the kinetic release of CHX from the DDS: stage I, hydration of MS; stage II, first burst of CHX release; stage III, plateau; and stage IV, second burst of CHX release.

Equations were calculated to characterize the kinetic release of both drugs in the system. The Korsmeyer–Peppas equation provided the best fit for describing the kinetic release of CHX and DXT. In the MS-CHX system, the constant (c) for this equation was found to be c = 0.698, indicating that the transport of CHX in the PLGA-MS followed a non-Fickian diffusion mechanism. Conversely, the c-value for the HG-DXT system was c = 0.358, indicating normal Fickian diffusion of DXT through the HG polymer. When evaluating the MS-CHX/HG-DXT DDS, the release behavior changed. For CHX, the c-value was >1, suggesting a super case II transport mechanism. Additional data and the total amount of drugs released from the system are presented in Table 4. The plots depicting the kinetic raw data and the percentage of drug release are shown in Figure 6. The combination of HG-DXT:MS:CHX in a 2:1 ratio exhibited the most promising performance for potential clinical applications. In this proportion, the DDS displayed an initial burst release of DXT from approximately 0.5 to 72 h, which is desirable for controlling postoperative pain in surgical wounds. Additionally, CHX began to release from the system at around 120 h, remaining constant for approximately 696 h. While this release behavior may vary under surgical conditions, the duration of CHX release could be valuable in preventing surgical site infections.

## 4. Discussion

The objective of this study was to design, synthesize, and characterize a dual drug delivery system (DDS) for the controlled local release of DXT and CHX. This DDS was specifically engineered to address the needs of endodontic microsurgical cases, aiming to improve the management of postoperative pain and prevent SSI.

In this study, CHX-loaded microspheres were synthesized (referred to as MS-CHX) demonstrating stability at body temperature. Various characterization methods were employed to confirm the presence of CHX in the MS-CHX group. Previous studies have examined the release of CHX from different systems [23,24,25,26,27]. Priyadarshini et al. reported the presence of aromatic amine groups (C=N) in vibrational bands of 1668 cm^−1^ and 1598 cm^−1^ [25,27]. In the present report, amine groups were detected at bands of 1521 cm^−1^ and 1491 cm^−1^. Upon consulting the BIO-RAD^®^ spectra database, characteristic peaks of CHX at approximately 1530 cm^−1^ and 1490 cm^−1^ were found, closely resembling the amine peaks of CHX. The slight variance between the peaks reported by Priyadarshini and those observed in present study could be attributed to the use of CHX diacetate salt in the Priyadarshini’s study. The presence of these peaks in the MS-CHX groups strongly indicates the successful incorporation of CHX within the microspheres. This finding was further supported by calorimetric curves, which suggested the presence of the drug in the loaded microspheres. Finally, the presence of CHX was confirmed through antibacterial effects and the MTS conducted on MS-CHX. These results provide additional validation of the successful incorporation and release of CHX from the microspheres.

Regarding the antimicrobial evaluation, Priyadarshini et al. conducted a study using CHX-loaded nanoparticles against *E. faecalis*, *S. salivarius*, and *S. mutans*. It is worth noting that all experimental groups demonstrated inhibition zones. However, the authors did not provide details on the method used to incorporate the nanoparticles into the paper disk [27]. In a separate study by Chen et al., the antimicrobial effect of their system against *S. aureus* was evaluated. They quantified the amount of CHX after 1 day using 10 mg of microspheres dissolved in 2 mL of PBS, showing that a significant antimicrobial effect for CHX-loaded microspheres was obtained, whereas no such effect was observed for the MS-Blank group [26]. Jiang et al. also reported the antimicrobial effect of their system against *P. aeruginosa*, with a release time spanning 4 to 8 weeks [24]. In our study, the microspheres are designed to release approximately 20,037.47 µg of CHX. These microspheres were dissolved in 0.5 mL of water, resulting in a final CHX concentration of approximately 4%, assuming the complete release of CHX from the microspheres.

In the cytotoxicity assay, Priyadarshini reported cell viability exceeding 80% in the experimental groups. They used dental pulp stem cells in their study, exposing them to concentrations of 25, 50, and 75 µg/mL of nanoparticles. However, it is important to note that they did not specify the exact quantity of CHX to which the cells were exposed [27]. Chen et al. evaluated the impact on cell proliferation in vitro using the MTT assay with a 3T3 cellular line. They replaced 100 µL of the culture media with the same volume of the suspension of CHX-loaded microspheres in their experiment, concluding that cell viability remained unaffected by the microspheres. It is worth mentioning that the lower CHX concentration and the incorporation of bFGF into the system, aimed at enhancing cell viability, make it challenging to directly compare their results to ours [26]. Jing et al. [24] assessed the cytotoxicity of their system using the MTS with 3T3 fibroblast cells. They suspended 10 mg of CHX-loaded microspheres in 1 mL of PBS at 37 °C, replacing the supernatant every week for up to 8 weeks to expose the cells for 48 h. The authors reported non-significant cytotoxicity of the microspheres, but this conclusion was drawn after comparing them to MS-Blank controls. In contrast, MS-CHX exhibited a cytotoxic effect against osteoblast cells in the MTS, with an approximate CHX concentration of 3963.33 µg/mL, based on the theoretically loaded CHX. The MS showed toxicity from 0.5 h to 7 days of evaluation. It is noteworthy that CHX did not produce cytotoxic effects in concentrations ranging from 0.0025 µg/mL to 0.02 µg/mL, as previously reported [28]. As shown, the concentration of microspheres was higher than these quantities. Interestingly, Jiang’s study reported a CHX release concentration of over 50 µg/mL within 10 days, a concentration previously considered cytotoxic. However, their reported results contradicted this expectation [24]. The real cytotoxic and biological response of the proposed delivery systems must be evaluated in further in vivo models.

While the literature contains only a limited number of reports on DDS designed for the controlled release of DXT, the utilization of PEG in hydrogel synthesis is extensively documented. The use of PEG for molecule release offers numerous advantages, including non-toxicity, biocompatibility, a wide range of available molecular weights (enabling different physical presentations), and chemical versatility [29,30,31]. These unique characteristics have enabled the creation of various DDS incorporating PEG. Depending on the cross-linkers employed, the molecular weight, and the combination with other polymers, hydrogels can exhibit diverse physical and chemical properties, leading to versatile release behaviors [30]. The biocompatibility of HG-DXT, as determined through the MTS, was clear. These findings align with numerous reports where PEG hydrogels have been successfully used in tissue engineering [32,33]. Previously, the cytotoxic effects of local DXT administration have been documented. Sagir et al. investigated the cytotoxic impact of locally administering 36.9 mg/mL of DXT to a chondrocyte cell line, employing 4.112 mg of DXT. Their study revealed that this concentration exhibited cytotoxic effects over various time intervals on these cells [34]. The present research showed that the maximum amount of DXT released was 2.5 mg (assuming complete drug release during the evaluation period). Considering that this hydrogel was designed for the controlled release of DXT over specific time intervals, the non-cytotoxic results can be attributed to reduced exposure to the drug. These results are particularly promising for the localized application of these DDS. Moreover, the release of CHX from the DDS proved more effective than that from the MS. The incorporation of MS-CHX into the HG system enhanced the kinetic release of CHX, particularly in the 2:1 proportion (HG-DXT:MS-CHX). This proportion is theoretically non-cytotoxic and holds promise for localized applications. However, further in vivo studies are essential for confirming these observations.

The thermal characteristics of the MS-CHX/HG-DXT DDS are a reflection of the individual systems’ thermal behavior, and the glass transition temperature of the MS is comparable with previous reports [35], ranging from 25.98 to 47.66 °C. This behavior corresponds to the change in the proportion of MS-CHX and HG-DXT. Notably, as the quantity of MS-CHX increases, the melting point shifts to the right. An important observation is the absence of well-defined endothermal peaks. The asymmetrical and irregular endothermal signals in this combination suggest a strong interaction between the two polymers used (PEG and PLGA) and a favorable incorporation of the drugs. Furthermore, it is important to note that once the entire DDS is prepared, it exhibits an endothermic signal at a temperature lower than the individual signals observed for MS-CHX and HG-DXT. Given that this behavior occurs near body temperature but remains stable below 30 °C, it suggests a promising release profile under physiological conditions. Nevertheless, this assumption must be validated in an in vivo model.

The reported concentration values of CHX for different DDS vary among authors. Priyadarshini et al. reported drug-loading values ranging from 10.49% to 19.49% [27]. In contrast, Chen et al. in their study reported a CHX loading of 1.42% to 1.85%, utilizing core–shell microspheres where CHX was loaded on the surface of the PLGA-MS [26]. In the present study, the CHX content within the MS was found to be 5.3%. The kinetic release of CHX from the MS may involve various mechanisms associated with PLGA degradation and erosion kinetics. In PLGA systems, drug release can be explained by a combination of phenomena, including diffusion through the polymer matrix, diffusion through aqueous pores, and dissolution concurrent with polymer dissolution.

For highly water-soluble drugs, such as CHX, diffusion through aqueous pores plays a significant role in drug transport [36]. In the case of MS-CHX, four distinct stages in the kinetic release were observed (Figure 6). The first stage is associated with the hydration process of the microspheres. The presence of CHX, as obtained by MS-CHX, becomes evident when it comes into contact with water. This was confirmed by the antibacterial effect observed in the disk diffusion test and the cytotoxic evaluation after 30 min of stirring. Following the hydration process, the PLGA chains begin to depolymerize, primarily due to hydrolysis occurring in the ester linkages. This hydrolysis initially proceeds non-catalytically throughout the polymer bulk, with the autocatalytic hydrolysis reaction becoming more prominent later [37]. As the hydrolysis progresses, the dissolution of small acid oligomers and monomers (such as lactic and glycolic acids) into the aqueous medium increases. This process hinders mobility in certain regions of the microspheres and generates an acidic microenvironment inside them [37]. The PLGA-based system exhibits autocatalytic degradation, with a low-pH environment being critical for this process [38]. The influence of pH has been previously studied and summarized by Ford Versypt et al. [37], who reported the formation of a low-pH microenvironment localized within the MS when using a buffered medium. These studies also identified additional factors contributing to autocatalytic behavior, including MS size, porosity, and the bulk degradation method. They suggest that a well-characterized degradation and erosion profile of PLGA MS can enhance drug release by supporting a triphasic pattern: an initial burst, a sustained release phase, and a secondary burst driven by internal autocatalysis. A similar release profile was observed in the present study, where HPLC-grade water was used as the medium to evaluate the kinetic release of two water-soluble drugs: DXT and CHX. Notably, Fu et al. [39] also did not report pH changes in the external medium, supporting the assumption that regular media replacement helps prevent the acidification of the release environment. Therefore, based on the previous literature and present findings, the use of HPLC water does not appear to significantly alter the drug release kinetics. Nevertheless, future studies will aim to validate and further characterize the drug delivery system (DDS) in animal models, incorporating various media and induced pH conditions.

After reaching a plateau, the lactic and glycolic monomers are gradually released from the microspheres. These molecules can contribute to lactate-induced angiogenesis, recruitment of endothelial progenitor cells, and vasculogenesis, as demonstrated in previous in vitro studies [40]. PLGA has been shown to significantly increase lactate levels in plasma in mouse models and can be considered for supplying lactate in wound treatment [41]. The final stage of CHX release is associated with a second burst. This behavior corresponds to the release of catalyzed monomers of PLGA, along with CHX. However, it is important to note that this stage may not be advantageous in a clinical setting when considering the microspheres alone. The amount of CHX released during this second burst, which occurs between 600 and 800 h (Figure 6), may potentially induce a toxic effect in tissues. As previously reported, concentrations exceeding 53 µg/mL can be problematic [42].

The kinetic release of CHX exhibited non-Fickian diffusion behavior, a process that occurs in a solid system when the diffusion temperature condition is below the glass transition temperature of the polymeric matrix [43]. The Tg of MS-CHX was determined to be 50 °C, while the release conditions were maintained at 37 °C. This disparity in temperatures explains the non-Fickian diffusion behavior observed. In contrast, the release of DXT from HG-DXT demonstrated an ideal pattern for clinical applications. DXT was released within the first 72 h, which is crucial for managing postoperative pain, especially during the initial stages when it tends to increase [44]. The release of DXT commenced immediately and maintained a favorable rate, especially within the first 200 h. This release profile aligns with the importance of effectively controlling pain in endodontic microsurgery, where pain is particularly significant during the first day and gradually diminishes in the subsequent days [3]. Notably, the DXT kinetic release followed a Fickian diffusion pattern, indicating that the polymeric matrix’s glass transition temperature was lower than the release protocol conditions. Specifically, the Tg of HG-DXT was found to be 36.29 °C, causing the hydrogel to transition from a gel-like state to a more fluid state. Furthermore, when the combination of MS-CHX and HG-DXT was in a 1:2 proportion, the kinetic release of both CHX and DXT exhibited behavior similar to that of the individual systems. In this combination, the system released DXT within the first 72 h and CHX after approximately 120 h, continuing up to around 600 h (Figure 6).

The perspectives for the clinical utilization of this DDS are highly promising. To put the concentration levels into context, the effective dose of DXT in plasma via oral administration is approximately 3.71 µg/mL when patients consume a tablet or capsule containing 25 mg of DXT [45] while the reported toxic concentration of DXT stands at 2056 µg/mL [34]. In DDS, the maximum concentration reached was approximately 50 µg/mL; this indicates that the system maintains DXT levels well within a safe range. On the other hand, the action mechanism of CHX is dose-dependent. Concentrations ranging from 0.02% to 0.06% exhibit bacteriostatic activity, whereas higher concentrations (>0.12%) act as bactericidal agents. The minimum inhibitory concentration (MIC) for periodontopathogens is reported to be 12.72 µg/mL [46], and the MIC90 for Staphylococcus aureus falls in the range of 2.5 to 5 µg/mL. However, it is worth noting that at these concentrations, CHX can produce cytotoxic effects [47]. Conversely, in some reports, CHX does not produce cytotoxic effects at lower doses, ranging from 0.0025 µg/mL to 0.02 µg/mL [24]. The cytotoxic effect appears at concentrations of 0.005% (53 µg/mL) in another cell line, underscoring the variability in findings across studies [42]. Moreover, available reports suggest beneficial effects of CHX in the healing of surgical wounds. When compared to other antibacterial solutions in dentistry, CHX exhibits a lower cytotoxic effect [47]. The concentration of CHX in the DDS surpasses the MIC90 for *S. aureus* [17], spanning from 72 h (in the MS-CHX alone) to 200 h (in the DDS MS: HG 1:2). While this higher concentration may raise concerns about cytotoxicity (as evaluated previously), it is important to note that the amount of CHX loaded during the synthesis process can be adjusted to achieve the optimal concentration for clinical applications. These results will be validated in further experiments. One of the limitations of this research is the lack of validation of the final DDS on animal models that may offer valuable information about possible clinical behavior. Also, additional experiments to evaluate the stability of the systems are needed in further studies.

Another intriguing avenue for exploration is the proposed analgesic effect of local CHX and its potential additive and synergistic effects when combined with DXT [48,49]. If these results can be replicated in human models, the antiseptic-analgesic DDS may represent a promising therapeutic alternative.

## 5. Conclusions

In the present study, it was possible to synthesize and characterize a drug delivery system comprising PLGA microspheres loaded with chlorhexidine gluconate and a PEG hydrogel loaded with dexketoprofen trometamol. Notably, the DDS with a 2:1 ratio (HG-DXT: MS-CHX) exhibited a highly favorable kinetic release profile for both molecules. The kinetics reveal a rapid release of DXT above therapeutic concentration for at least 200 h, which favors the postoperative pain control. Additionally, the sustained release of CHX at antibacterial concentrations for up to 700 h confers a lasting duration to prevent possible SSIs, indicating its promising potential for clinical applications.

## Figures and Tables

**Table 1:** Summary of the development of DDS (Experimental groups).

Abbreviature	Description	Content	Manufacturer
CHX	Chlorhexidine	Chlorhexidine digluconate solution 20% in H_2_O	Sigma-Aldrich
PLGA	Poly(lactic-co-glycolic acid)	50:50; Molecular weight range ∼66,000–110,000	Sigma-Aldrich
MS-Blank	Empty microspheres (MS)	Only PLGA	NA
MS-CHX	CHX-Loaded microspheres	PLGA + CHX	NA
DXT	Dexketoprofen Trometamol	Dexketoprofen Trometamol powder	STEIN labs
PEG	Polyethylene glycol	PEG (MW: 400 and 4000)	Sigma-Aldrich
HG-Blank	Empty Hydrogel (HG)	Only PEG	NA
HG-DXT	DXT-loaded hydrogel	PEG + DXT	NA
DDS	Drug delivery system	MS-CHX + HG-DXT	NA

**Table 2:** Antibacterial evaluation of the MS (8 days of stirring).

Groups (*n* = 3)	Strain	Inhibition Zone (mm) Mean (sd)	*p*-Value *
MS-CHX	*E. faecalis*	15.6 (0.53)	*p* < 0.05
MS-Blank		6 (0)	
CHX 2%		15.5 (0.5)	
Sensi-disk		20.03 (0.06)	
MS-CHX	*C. albicans*	16.3 (1.42)	*p* < 0.05
MS-Blank		6 (0)	
CHX 2%		27.5( 1.2)	
Sensi-disk		25.6 (0.58)	
MS-CHX	*E. coli*	14.23 (0.45)	*p* < 0.05
MS-Blank		6 (0)	
CHX 2%		15.5 (0.5)	
Sensi-disk		28.1 (0.1)	
MS-CHX	*S. aureus*	17.83 (1.61)	*p* < 0.05
MS-Blank		6 (0)	
CHX 2%		27.5 (1.32)	
Sensi-disk		25.67 (0.2)	

* One-way ANOVA.

**Table 3:** Cytotoxicity evaluation of the MS, HG and DDS.

**Cytotoxicity Evaluation of the MS**		
Groups (*n =* 5)	Cell Viability (%) Mean (SD)	Kruskal–Wallis Test
MS-Blank	172 (14.2)	*p* < 0.05
MS-CHX (0.5 h)	29.2 (5.35)	
MS-CHX (2 h)	19 (1.94)	
MS-CHX (24 h)	17.3 (1.49)	
MS-CHX (48 h)	18.7 (1.16)	
MS-CHX (8 days)	19 (1.8)	
CHX 0.2%	29 (2.87)	
**Cytotoxicity evaluation of the HG**		
Groups (*n =* 5)	Cell viability (%) Mean (SD)	ANOVA test
HG-Blank	82.4 (9.15)	*p* > 0.05
HG-DXT (0.5 h)	83.4 (25.2)	
HG-DXT (1 h)	103 (1.94)	
HG-DXT (3 h)	87.7 (24.1)	
HG-DXT (6 h)	81.3 (10.5)	
HG-DXT (8 h)	79.2 (6.38)	
DXT 2.5 mg	68 (19.2)	
**Cytotoxicity evaluation of the MS, HG and DDS at direct contact with cells**		
Groups (*n =* 5)	Cell Viability (%) Mean (SD)	ANOVA test
MS-Blank	89.2 (1.77)	*p* < 0.05
HG-Blank	74.8 (17.0)	
HG-Blank+MS-Blank (2:1)	132 (6.41)	
MS-CHX	53.8 (3.71)	
HG-DXT	69.2 (4.02)	
HG-DXT+MS-CHX (2:1)	16.2 (2.13)	

**Table 4:** Kinetics release and quantification of CHX and DXT from the DDS.

**Total Amount of Drug Release in the DDS**							
Group	Mean of CHX Content in µgr (% of Drug Release)			Mean of DXT Content in µgr (% of Drug Release)			*n*
MS-CHX	~536.74 µgr (~91.57%)			NA			3
HG-DXT	NA			~242.35 µgr (96.94%)			3
MS:HG 1:1	~246.26 µgr (~84.06%)			~60.65 µgr (48.52%)			3
MS:HG 1:2	~156.99 µgr (~80.38%)			~150.39 µgr (90.24%)			3
**Detection points in the kinetic release of CHX and DXT**							
Group	Chlorhexidine gluconate			Dexketoprofen trometamol			*n*
	**FS**	**LS**	**Rate**	**FS**	**LS**	**Rate**	
MS-CHX	72 h	840 h	0.698 µgr/h	NA	NA	NA	3
HG-DXT	NA	NA	NA	0.5 h	336 h	0.722 µgr/h	3
MS:HG 1:1	120 h	768 h	0.38 µgr/h	24 h	624 h	0.101 µgr/h	3
MS:HG 1:2	120 h	696 h	0.272 µgr/h	0.5 h	240 h	0.627 µgr/h	3
**Kinetic models for CHX release**							
Group		Zero Order		First Order		Korsmeyer-Peppas	
		R^2^	K_µgr·h_^−1^	R^2^	K_h_^−1^	R^2^	c
MS-CHX		0.969	0.135	0.976	−0.0008	0.988	0.698
MS:HG 1:1		0.974	0.093	0.883	−0.0007	0.996	1.668
MS:HG 1:2		0.949	0.089	0.783	−0.0007	0.997	2.128
**Kinetic models for DXT release**							
Group		Zero Order		First Order		Korsmeyer-Peppas	
		R^2^	K_µgr·h_^−1^	R^2^	K_h_^−1^	R^2^	c
HG-DXT		0.889	0.883	0.961	−0.007	0.989	0.358
MS:HG 1:1		0.941	0.097	0.92	−0.0005	0.979	0.598
MS:HG 1:2		0.921	0.699	0.947	−0.0043	0.994	0.398

*n =* number of samples, FS = first signal detected by HPLC, LS = last signal detected by HPLC, c = constant of the model (release exponent), K = release rate.

## Data Availability

Data is contained within the article.

## Abbreviations

The following abbreviations are used in this manuscript:

DDS	Drug delivery system
PLGA	Poly(lactic-co-glycolic acid
MS-CHX	Microspheres loaded with chlorhexidine
PEG	Polyethylene glycol
HG-DXT	Hydrogel containing dexketoprofen
DSC	Differential scanning calorimetry
TGA	Thermogravimetric analysis
SEM	Scanning electron microscopy
FTIR	Fourier transform infrared spectroscopy
EDS	Energy-dispersive X-ray spectroscopy
HPLC	High-performance liquid chromatography
CHX	Chlorhexidine digluconate
DXT	Dexketoprofen
COXs	Cyclooxygenases
NSAIDs	Non-steroidal anti-inflammatory drugs
SSI	Surgical site infections
MS	Microspheres
DCM	Dichloromethane
PVA	Polyvinyl alcohol
HG	Hydrogel
PTFE	Polytetrafluoroethylene
Tp	Inflection point
Tg	Glass transition
MIC	Minimum inhibitory concentration
